# Health economic evaluation of a nurse-led care model from the nursing home perspective focusing on residents’ hospitalisations

**DOI:** 10.1186/s12877-022-03182-5

**Published:** 2022-06-09

**Authors:** Jana Bartakova, Franziska Zúñiga, Raphaëlle-Ashley Guerbaai, Kornelia Basinska, Thekla Brunkert, Michael Simon, Kris Denhaerynck, Sabina De Geest, Nathalie I. H. Wellens, Christine Serdaly, Reto W. Kressig, Andreas Zeller, Lori L. Popejoy, Dunja Nicca, Mario Desmedt, Carlo De Pietro

**Affiliations:** 1 Department Public Health, Institute of Nursing Science, Faculty of Medicine, University of Basel, Basel, Switzerland; 2 Institute of Biophysics and Informatics, 1St Faculty of Medicine, Charles University, Prague, Czech Republic; 3 University Department of Geriatric Medicine FELIX PLATTER, Basel, Switzerland; 4 Department of Public Health and Primary Care, Academic Centre for Nursing and Midwifery, KU Leuven, Louvain, Belgium; 5 Department of Public Health and Social Affairs, Directorate General of Health, Canton of Vaud, Lausanne, Switzerland; 6 La Source School of Nursing, HES-SO University of Applied Sciences and Arts Western Switzerland, Lausanne, Switzerland; 7 serdaly&ankers Conches, Switzerland; 8 Faculty of Medicine, University of Basel, Basel, Switzerland; 9 Centre for Primary Health Care, University of Basel, Basel, Switzerland; 10 The University of Missouri, Sinclair School of Nursing, Columbia, US; 11 Institute of Epidemiology, Biostatistics and Prevention, University of Zürich, Conches, Switzerland; 12 Foundation Asile Des Aveugles, Lausanne, Switzerland; 13 Department of Business Economics, Health and Social Care, University of Applied Sciences and Arts of Southern Switzerland, Lugano, Switzerland

**Keywords:** Health economics, Cost-effectiveness analysis, Time-driven activity-based costing, Implementation science, Nurse-led care model, Nursing home, Hospitalisation

## Abstract

**Background:**

Health economic evaluations of the implementation of evidence-based interventions (EBIs) into practice provide vital information but are rarely conducted. We evaluated the health economic impact associated with implementation and intervention of the INTERCARE model—an EBI to reduce hospitalisations of nursing home (NH) residents—compared to usual NH care.

**Methods:**

The INTERCARE model was conducted in 11 NHs in Switzerland. It was implemented as a hybrid type 2 effectiveness-implementation study with a multi-centre non-randomised stepped-wedge design. To isolate the implementation strategies' costs, time and other resources from the NHs’ perspective, we applied time-driven activity-based costing. To define its intervention costs, time and other resources, we considered intervention-relevant expenditures, particularly the work of the INTERCARE nurse—a core INTERCARE element. Further, the costs and revenues from the hotel and nursing services were analysed to calculate the NHs' losses and savings per resident hospitalisation. Finally, alongside our cost-effectiveness analysis (CEA), a sensitivity analysis focused on the intervention's effectiveness—i.e., regarding reduction of the hospitalisation rate—relative to the INTERCARE costs. All economic variables and CEA were assessed from the NHs' perspective.

**Results:**

Implementation strategy costs and time consumption per bed averaged 685CHF and 9.35 h respectively, with possibilities to adjust material and human resources to each NH’s needs. Average yearly intervention costs for the INTERCARE nurse salary per bed were 939CHF with an average of 1.4 INTERCARE nurses per 100 beds and an average employment rate of 76% of full-time equivalent per nurse. Resident hospitalisation represented a total average loss of 52% of NH revenues, but negligible cost savings. The incremental cost-effectiveness ratio of the INTERCARE model compared to usual care was 22′595CHF per avoided hospitalisation. As expected, the most influential sensitivity analysis variable regarding the CEA was the pre- to post-INTERCARE change in hospitalisation rate.

**Conclusions:**

As initial health-economic evidence, these results indicate that the INTERCARE model was more costly but also more effective compared to usual care in participating Swiss German NHs. Further implementation and evaluation of this model in randomised controlled studies are planned to build stronger evidential support for its clinical and economic effectiveness.

**Trial registration:**

clinicaltrials.gov (NCT03590470)

## Background

Increasing numbers of residential long-term care facilities are implementing evidence-based interventions (EBIs) [1–3]. However, while health economic evaluations of implementations are vital regarding large-scale rollout, they remain scarce [4–6]. The current paper evaluates the health economic aspects of an EBI to reduce hospitalisations of nursing home (NH) residents—the INTERCARE nurse-led care model (Nurse-led model in Swiss nursing homes: improving INTERprofessional CARE for better resident outcomes) [7]. To maximise acceptance and feasibility, the INTERCARE study followed an implementation science approach: after a rigorous contextual analysis [8, 9], six bundled evidence-based core interventions were tailored to the target context and systematically introduced [7]. The clinical effectiveness was measured by an objective outcome: change in the number of hospitalisations [10]. This paper focuses on the costs, time and other resources arising from the implementation strategies and intervention.

In healthcare, EBIs are initially driven by research and financed by ad-hoc grants. When clinical outcomes indicate their effectiveness, a viable introduction and further sustainable rollout in real-life settings often hinge on questions of time, costs and other resources. Despite evidence that EBIs can be cost-effective over time [11, 12], then, their implementation is challenged by the absence of detailed information about costs related to adopting new practices, including information that aids logistical decisions, which would likely increase various stakeholders' willingness to implement EBI [13]. Therefore, to maximise stakeholder buy-in, we aimed to determine the INTERCARE model's health-economic efficiency by analysing its impact on the participating NHs’ consumed costs, time and resources.

The INTERCARE study developed and evaluated the INTERprofessional nurse-led CARE model (INTERCARE) that was comprised of six core elements, including an INTERCARE nurse in an expanded role (see Methods section for detailed information). The overall aim of INTERCARE was to reduce unplanned transfers from NHs to hospitals in Switzerland's German-speaking region [7].

The INTERCARE study is a Hybrid Type 2 effectiveness-implementation study: it focuses equally on effectiveness (i.e., reductions in unplanned hospitalisations) and implementation outcomes (e.g., fidelity, feasibility, cost) [14]. Several bundled implementation strategies were used to support NHs in their uptake of the INTERCARE nurse-led care model. Working from the NHs’ perspective, this paper reports on our health economic evaluation of the INTERCARE study with regard to: (a) costs, time and resources arising from the NHs' participation in several of the study's implementation strategies; and (b) the economic investment required when introducing the core elements into practice. Additionally, we assessed revenue losses caused by residents’ hospitalisations. Finally, we calculated the cost-effectiveness of the INTERCARE intervention for participating NHs vis à vis resident hospitalisation. Our health economic evaluation aims to tackle the above-mentioned barriers to facilitate a scale-up from the funded research phase to a feasible integration into cost-effective and otherwise sustainable routine practice.

## Methods

### Aims

For the health-economic analysis described in this paper, the INTERCARE study has four purposes: (1) to implement the INTERCARE nurse-led care model while providing an overview of the participating NHs’ costs, time and resources used; (2) to evaluate participating NHs’ intervention costs, time and resources, particularly the INTERCARE nurse as the main cost factor of the INTERCARE model's six core elements; (3) to evaluate the NHs’ potential losses, savings and revenues resulting from hospitalisation; and (4) working from the NHs’ perspective, focusing on avoided hospitalisations, to compute the INTERCARE intervention's cost-effectiveness versus usual (i.e., pre-intervention period) care.

### Study design and periods

**Fig. 1:**
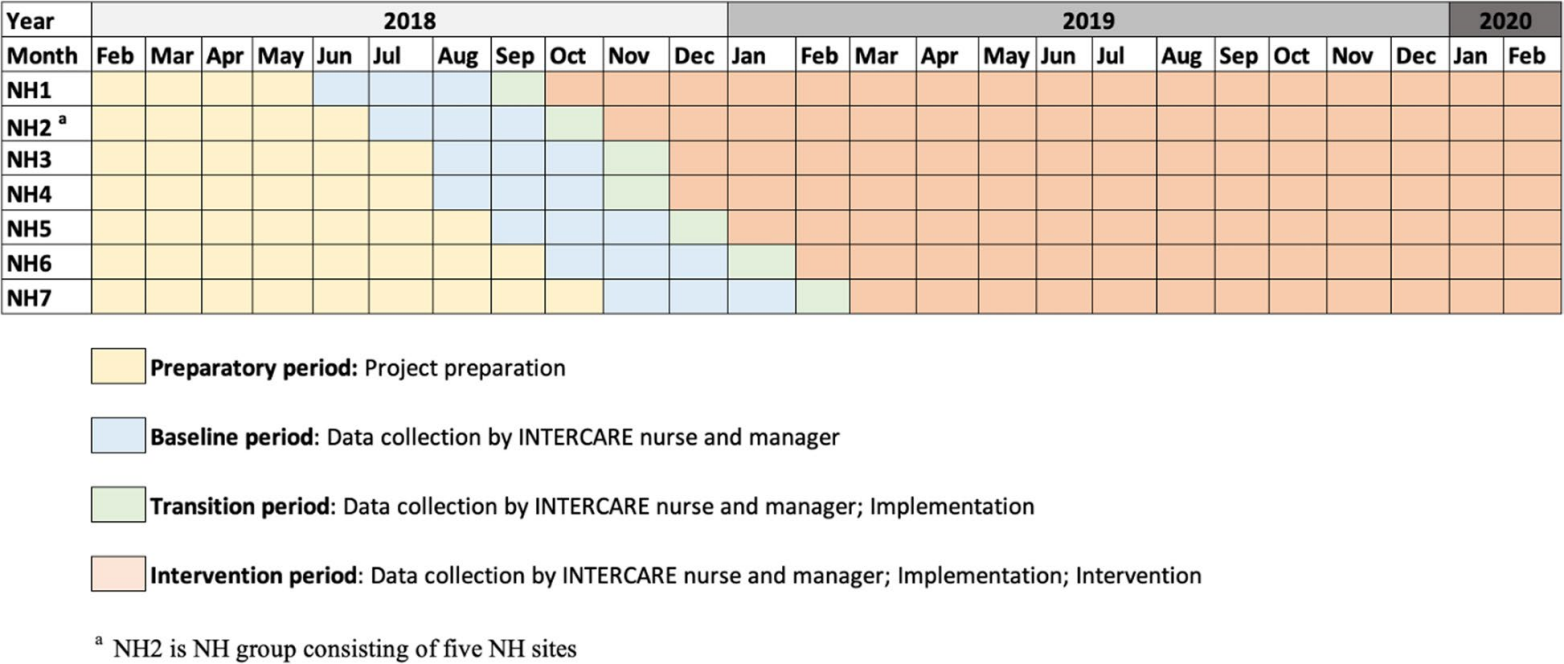
Nonrandomised stepped-wedge design and the periods of the INTERCARE study

A non-randomised stepped-wedge design was used. This is described in detail in the study protocol [7] (Fig. 1). Clinical and economic data were collected from June 1, 2018 to February 29, 2020. INTERCARE is registered with clinicaltrials.gov (Protocol Record NCT03590470).

Each NH started with a preparatory period (the time between approval for participation in INTERCARE and start of data collection), followed by three months of baseline measurement (pre-implementation data collection) and a one-month transition period (to address possible timing problems with the model's start and to start the implementation). We then began the intervention period (continuation of implementation, beginning of intervention), which lasted until the end of the trial period, February 29, 2020 (Fig. 1). Implementation of the model started at the first NH in September 2018. Then, each month, 1–2 NHs were started sequentially (Fig. 1).

### Sample

Six single sites and one five-NH group, all from the German-speaking part of Switzerland, were selected. NHs were included if they had 60 or more long-term care beds and had recorded at least 0.8 hospitalisations per 1′000 resident days over the previous year. All long-term care residents who provided informed consent were included in the data collection. Further inclusion and exclusion criteria for the NHs and residents can be found in the study protocol [7]. The NHs' characteristics were previously described by Zúñiga et al. [10].

### Core intervention elements of the INTERCARE model

The INTERCARE nurse-led care model consists of a bundle of six core intervention elements: strengthening interprofessional collaboration; using the expanded clinical role of an INTERCARE nurse; conducting comprehensive geriatric assessments; administering evidence-based tools to strengthen communication and reflect on unplanned hospitalisations; applying advance care planning; and using data-driven quality improvement. This complex intervention is described in detail in the study protocol [7].

### Implementation strategy actions

In discussing implementation (strategy) actions, we follow Cidav et al.'s definition [15] (p. 4): “any discrete activity involving one or more resources—personnel and/or equipment” needed to deliver an implementation strategy.

Multiple implementation strategies on the levels of preparatory planning, continuous support, education/training, and quality management [7] were used (see Table 1). These were categorised according to The Expert Recommendations for Implementing Change (ERIC) compilation [16], and included, e.g., assessing NHs’ readiness for implementation, conducting preparatory leadership meetings to identify barriers and facilitators for model implementation, conducting ongoing INTERCARE nurse training within a blended learning curriculum, providing ongoing consultation and local technical assistance, and auditing/providing feedback during regular meetings or phone calls with NHs [7]. Table 1 describes the concrete actions (i.e., meetings, phone calls, training sessions) used to apply the implementation strategies.

**Table 1:** Implementation strategy actions and Implementation team

**IMPLEMENTATION STRATEGY ACTIONS**					
**What**	**When**	**Where**	**Length**	**Participants**	**Aim**
**Preparatory leadership meetings** **1**^**st**^ **2**^**nd**^ **3**^**rd**^	Preparatory period	Research group premises	8 h 3 h 3 h	NH director and/or director of nursing, INTERCARE nurse, project manager^a^ optional: home-based GP, financial director, data manager, unit manager^b^	-to assist with planning the implementation of core and peripheral components (see study protocol [7]) -to give support in the local tailoring and buy-in from co-workers -to work out the content of the new role and its added value for RNs -to identify barriers and facilitators -to answer questions
**Meeting with all NHs**	Intervention period	Research group premises	7 h		-to facilitate the exchange between NHs -to share the first results/insights of the exploratory study phase with contextual analysis -to discuss the implementation strategies -to discuss the necessary factors to obtain sustainable local implementation
**Leadership and INTERCARE nurses' meetings**	Transition period, Intervention period	NH	2 h per meeting; number of meetings differed between NHs depending on the start of the Transition period		-to discuss the implementation of the intervention elements -to discuss the primary and secondary clinical outcomes, implementation outcomes, implementation strategies -to answer questions
**INTERCARE nurses' training**	Preparatory period, Baseline period, Transition period, Intervention period	Research group premises, e-learning	390 h	INTERCARE nurse	-to prepare INTERCARE nurses to take over the clinical leadership roles and reduce the number of unplanned hospital admissions
**Phone calls**	Transition period, Baseline period, Intervention period	NH	twice a month with INTERCARE nurse for one hour	INTERCARE nurse	-to discuss the implementation of intervention elements within the responsibility of the INTERCARE nurse -to support blended learning in the context of the INTERCARE curriculum (whole educational program) -to discuss challenges, barriers and facilitators in the role of INTERCARE nurse
**Internal training and information events**	Preparatory period, Baseline period, Transition period, Intervention period	NH	Differ between NHs	Internal decision of NH	-to build a strong foundation for the implementation of the intervention elements -to establish realistic expectations and deliver cutting-edge practices -to empower teams through various educational activities -to coach about evidence-based instruments (STOP and WATCH, ISBAR, hospitalisation reflection tools) (see study protocol [7])
**Administration and internal coordination**	Preparatory period, Baseline period, Transition period, Intervention period	NH	Different between NHs	Internal decision of NH	-to ensure quality management -to regulate study coordination -to correct negative deviations and assure the accomplishment of plans -to indicate where coordination is still required and to make suitable changes
**IMPLEMENTATION TEAM**					
**Group**	**Name**		**Description**		
**Research group**	Implementation team (i.e., research team)		The research group included members from nursing, medicine, policy, and practice representing several institutions (university, university of applied sciences, cantonal public administration office, consulting firm, NH, hospital) and three language regions of Switzerland. The team developed and distributed educational materials (including guidelines, decision trees, handouts and PowerPoint presentations). All materials were made available on online learning platforms and/ or sent by email to support the implementation activities in the NHs.		
	Study coordinator (part of the Implementation team)		Two-weekly phone calls with INTERCARE nurses to discuss individual challenges during the implementation process and to give feedback, support them in their personal development by critically reflecting on their own and others' behaviours and skills and discussing challenges, as well as to ensure effective knowledge transfer.		
**NH group**	INTERCARE team		Study team within NH with the upper management members. The size and the other team members depend on NH organisation (INTERCARE nurse, physicians, unit leaders, etc.).		
	INTERCARE nurse (part of the INTERCARE team)		This role is one of the six INTERCARE intervention elements. At the same time, she/he was key in introducing other intervention elements into practice. On the one hand, the INTERCARE nurse is a core element of the intervention. Each NH employed at least one INTERCARE nurse to take over a clinical leadership role. The role aimed to support the care workers in complex clinical situations, facilitate interprofessional collaboration, collect resident's data and advance clinical practice. On the other hand, in this role, she/he was responsible for implementing the core element evidence-based instruments in the NHs while distributing education materials, providing hands-on coaching, answering questions, and supporting staff in the use of instruments. In some NHs, depending on the study team structure, they were also responsible for implementing the “Advance Care Planning” intervention element. INTERCARE nurses followed a 390-h blended learning curriculum to prepare for their role. This included eight modules: Clinical leadership; Communication; Comprehensive geriatric assessment / Advance care planning; Geriatric syndromes; Chronic conditions; Acute symptoms; Medication management; Data-driven quality improvement. Learning methods include: E-learnings, readings, self-evaluations, reflections, face-to-face meetings, supervision and exchange among participants. Where more than one but less than two full-time INTERCARE nurses were necessary, the NHs decided whether they would split the role into two with smaller percentages and unequal or evenly shared responsibility.		

^a^ if the INTERCARE nurse was not a project manager

^b^ unit manager only in a leadership meeting in the intervention period

### Variables and measurements

For the five-site NH group, costs, time, resources and revenues were reported overall as a group due to their joint overhead organisation. Their clinical and economic variables and measurements are similarly reported for the entire group rather than per site.

### Clinical

For the economic analysis, the primary clinical variable was the overall number of hospitalisations, i.e., admissions from the NH to an acute care setting for a planned or unplanned reason, with at least one overnight stay, excluding psychiatry referrals.

### Economic

All economic variables were assessed from the participating NHs' perspective. Overall costs, time and resources used to implement INTERCARE were calculated for the preparatory, transitional and intervention periods. Specific intervention costs, time and resources were calculated for the intervention period (Fig. 1). All costs are reported in Swiss Francs (CHF), thus no currency conversion was needed. The purchasing power parity of CHF to US dollar was 1.140 in 2020 and 1.109 in 2021 [17].

To define total costs, time and resources per implementation strategy action and per NH, we applied time-driven activity-based costing (TDABC) [15]. TDABC is a process-based micro-costing methodology that provides detailed cost data through process maps [18] and is well matched to implementation science’s focus on EBI uptake in healthcare [15, 18, 19]. The related template was prepared prospectively by including questions about main actions (what), temporality (when), actors (who), action frequency and unit duration (length) and actor wage rate. Eight discrete implementation strategy actions were defined and classified as personnel resources: (a) INTERCARE preparatory leadership meeting; (b) INTERCARE meeting with all NHs; (c) leadership and INTERCARE nurse meetings; (d) INTERCARE nurse training; (e) phone calls; (f) internal training and information events; (g) administration and (h) internal coordination. Points (a) to (e) were implementation strategy actions organised by the research group, with equal time offered to each NH (e.g., bi-weekly phone calls, bi-monthly leadership meetings), although the net time differed based on variations in the duration of the intervention period. Points (f) to (h) were implementation strategy actions organised independently by the NHs for locally tailored implementation of the core elements. Variations reflected differences in their internal structures and processes. Information on non-personnel resources (e.g., essential travel to in-person meetings; equipment, technology and other materials required to perform the actions) was also collected. We populated the process maps by relevant data recorded by managers throughout study via survey.

As the analysis was from the NH perspective, it did not consider our group's research-specific expenditures (costs, time or other resources), e.g., for data preparation, attending research team meetings or developing and distributing educational material.

For the evaluation of the intervention costs, time and resources, only additional work of INTERCARE nurses was considered. Most core intervention elements were integrated into ongoing NH processes, e.g., using a structured assessment for resident pain. On the other hand, since the INTERCARE nurse position required additional financing, it was considered a cost-intensive intervention element. The intervention costs were expressed as the average yearly INTERCARE nurse salary per bed during the intervention period; intervention time was expressed as average employment percentage per INTERCARE nurse; and intervention resources were given as average number of INTERCARE nurses per 100 beds. We assumed that all INTERCARE nurses had a 13^th^ month salary (standard remuneration practice in Switzerland), and that employment percentages and pay rates remained constant over the period and year, respectively. Additional bonuses were not included. As the intervention period varied between participating NHs (12–17 months), intervention costs were calculated per year.

Regard to residents’ stays, NHs' costs and revenues include “hotel services” and “nursing services.” Hotel services include all services for accommodation (e.g., furnished room, energy consumption, full board, laundry, shared use of the general infrastructure). As a general rule, this fee is paid by the resident. According to Swiss laws for long term care funding, the revenue for nursing services is based on a 12 care-level case-mix system measured with a resident assessment tool (e.g., The Resident Assessment Instrument–Minimum Data Set adapted for Switzerland). Each level adds 20 min of care per day (max. 240 min/day). This is covered by mandatory health insurance.

We calculated the revenues from nursing services per NH as simple average revenues over the 12 case-mix levels. Our calculation did not include the costs of special services, e.g., special palliative care, a residential group for people with dementia, dental treatment, medically indicated transport, surcharges for single rooms and apartments, food supplements not prescribed by doctors, or expenses for personal needs. We assume that special services are: i) billed directly to the resident based on consumption; ii) priced to cover the costs without any net loss or gain for the NH; and iii) supplied/provisioned in a way that does not imply fixed costs or revenues for the NH.

From the NHs' perspective, the hospitalisation generates an empty bed. This can have different impacts on costs and revenues. First, some costs will decrease (e.g., laundry), resulting in savings for the NH. We call them variable costs. Second, other costs will remain the same (e.g., contracts for maintenance of facilities). These are called fixed costs. Third, some revenues will decrease (e.g., reimbursements from health insurance), thus resulting in loss of revenue for the NH. We call these variable revenues. Last, some revenues will remain the same (e.g., if the NH continues to bill a co-pay for hotel services, also if the resident has been temporarily transferred to the hospital).

Furthermore, based on the regulations provided by the participating NHs, arrival and departure days were excluded as days of absence.

### Data collection

#### Clinical

Each NH's manager collected fully anonymised, routine overall hospitalisation resident stay data (date of hospitalisation, date of discharge) from their administrative software from January 2017 (i.e., seventeen to twenty-two months before the start of the INTERCARE study) to March 2020 (i.e., the month after the intervention period ended). The resident data from January 2017 to the start of the INTERCARE intervention period served as the input variables for the comparator group in the cost-effectiveness analyses. Due to data availability issues, only transfers with at least one overnight stay could be included. Ethical approval was granted by all ethics committees responsible for the participating NHs (EKNZ 2018–00501).

#### Economic

Using a self-developed questionnaire, we surveyed the managers of the participating NHs via e-mail to determine:

- Costs of implementing INTERCARE (i.e., investments in the eight implementation strategy actions; travel; material; staff salaries);
- The method of financing INTERCARE nurses, their number and employment percentage (at baseline, six and twelve months after baseline), their salaries (2018, 2019, 2020) and how those salaries were embedded in the overall salary structure;
- Costs and revenues associated with residents’ hospitalisations (variable and fixed costs, variable and fixed revenues, other related internal regulations).

We asked every NH for their price list of services provided to residents for the years 2017–2020 and relevant information about hospitalisations' impact on their revenues. If there were incongruences between the price list and NH managers' answers, valid data from the price list were inserted in the data collection form.

Questionnaires were emailed to NH managers, who consulted with their accounting staff. Each questionnaire contained a cover sheet describing the purpose of the data collection. NHs were assured that any report made available to the public would not contain any identifying information.

### Statistical analysis

To illustrate the intervention, implementation and hospitalisation costs, descriptive statistics were employed, reporting ranges, averages, means, standard deviations and percentages as appropriate. SAS 9.4. (SAS Institute, Cary, NC) was used for statistical analysis, and Microsoft Excel for graphic presentation of data.

In addition to detailing the costs of the intervention, implementation, and hospitalisations from the perspective of the NH, we also calculated the cost-effectiveness of the intervention. Our calculation of the incremental cost-effectiveness ratio (ICER) reflected the NHs’ differences in nursing days by including them in the calculation as follows:

$$\mathrm{effects},\;\mathrm{where}\;\mathrm{the}\;\mathrm{hospitalisation}\;\mathrm{rate}\;=\frac{\mathrm{number}\;\mathrm{of}\;\mathrm{hospitalisations}\;\mathrm{per}\;\mathrm{NH}\;\mathrm x\;\mathrm{per}\;\mathrm{month}\;\mathrm y\;}{\mathrm{number}\;\mathrm{of}\;\mathrm{nursing}\;\mathrm{days}\;\mathrm{per}\;\mathrm{NH}\;\mathrm x\;\mathrm{per}\;\mathrm{month}\;\mathrm y}\ast1000$$

and

$$\mathrm{costs},\;\mathrm{where}\;\mathrm{the}\;\mathrm{salary}\;\mathrm{rate}\;=\frac{\mathrm{salaries}\;\mathrm{of}\;\mathrm{INTERCARE}\;\mathrm{nurses}\;\mathrm{per}\;\mathrm{NH}\;\mathrm x\;\mathrm{per}\;\mathrm{month}\;\mathrm y}{\mathrm{number}\;\mathrm{of}\;\mathrm{nursing}\;\mathrm{days}\;\mathrm{per}\;\mathrm{NH}\;\mathrm x\;\mathrm{per}\;\mathrm{month}\;\mathrm y}\ast1000.$$

Thus, the ICER was measured as the increase in staff costs during the intervention period divided by the decrease of hospitalisation rate (hospitalisation rate through the intervention period minus hospitalisation rate before intervention). The choice to consider only the cost of the INTERCARE nurse is based on two reasons. On the one hand, it constitutes the largest incremental cost item of the intervention. On the other hand, the different billing rules adopted by NHs in the case of hospitalisation, make the net economic impact of hospitalisations vary and of little relevance to the analysis. As there is no other alternative to INTERCARE, the only difference between pre-intervention period (i.e., usual care) and intervention period is the intervention itself. Thus, the costs for usual care in our ICER are zero. We assumed that the costs and effects of the intervention occurred in the same year, thus we kept the discount at 0%.

To establish the robustness of the results (considering the uncertainty level) of our cost-effectiveness analysis (CEA), we performed a univariate sensitivity analysis. We modified the value of one base case variable at a time, recording the corresponding costs and effects. We modified number of nursing days and number of hospitalisations by ± 20% (as the sample ranges are widely influenced by the size of NH) and in the salary rate and hospitalisation rate before and after INTERCARE we used the ranges of our sample. The Guidelines for the Economic evaluation and other CEA literature supports the use of ± 20% for pragmatic modification of the base case variables where the range is not available or suitable [20–22].

For graphic presentation, we followed the recommendations of The Professional Society for Health Economics and Outcomes Research (ISPOR): a tornado diagram [23]. In this, the horizontal axis lists the outcomes; along the vertical, parameters are arrayed; bars represent the outcome range associated with each parameter’s range. The outcome point estimate corresponding to each base-case ICER value is indicated by a vertical line cutting through all horizontal bars. The longest bar (reflecting the parameter generating the widest uncertainty) is placed at the top and the other bars are arrayed in descending order of length [23].

## Results

### Implementation costs, time and resources

**Fig. 2:**
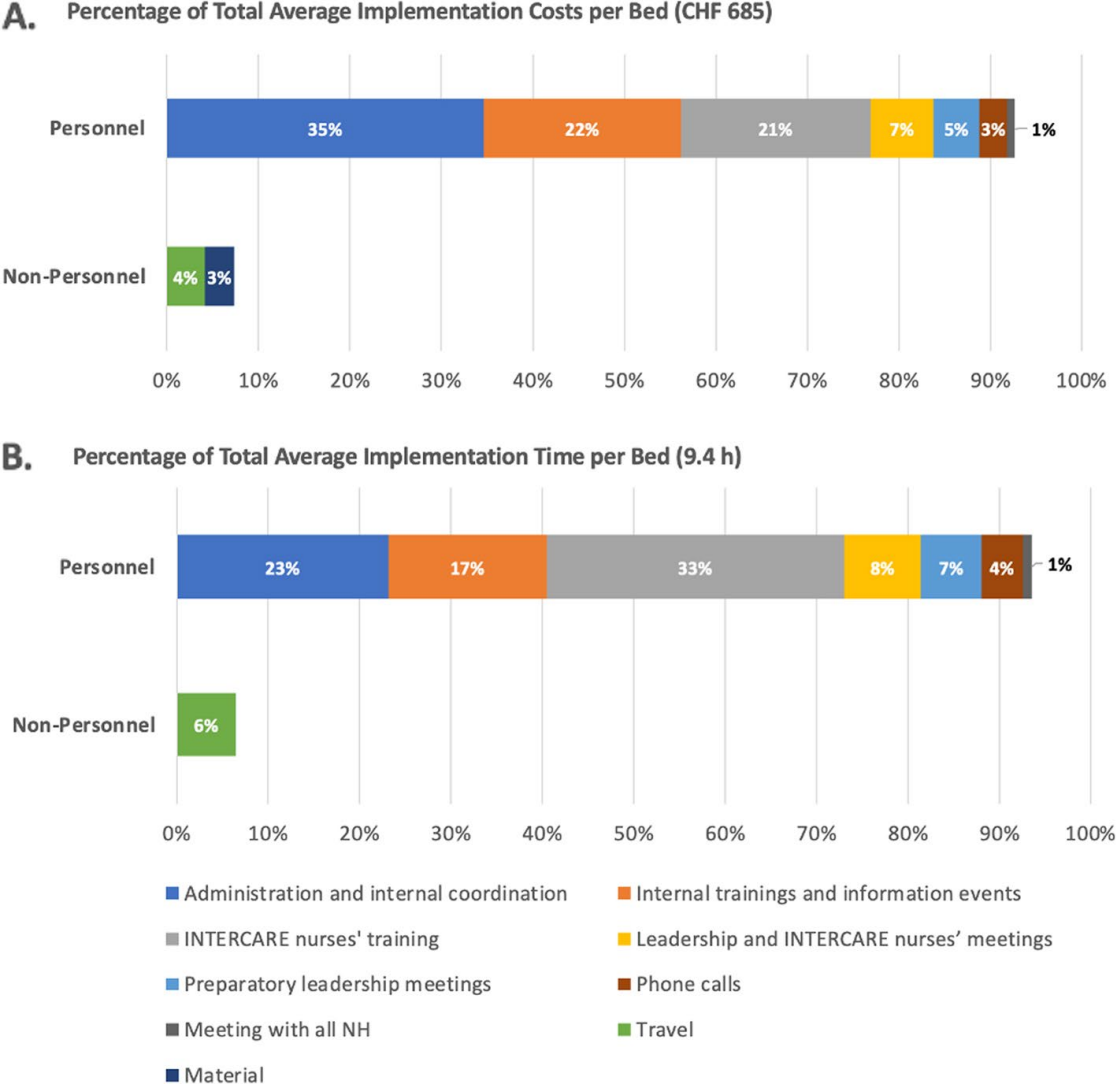
Composition of INTERCARE's implementation costs **A** and time **B**

The average total implementation costs per bed were 685CHF (range 110–1′591CHF); the average total implementation time per bed was 9.35 h (range 2.05–17.16 h). The most cost- and time-intensive areas were “Administration and internal coordination” and “Internal training and information events”, i.e., respectively, the NHs' necessary work to implement INTERCARE, and "INTERCARE nurse training". Across all NHs, these three areas constituted 78% (range 44–82%) and 73% (range 45–86%) respectively of the total costs and time of implementation. Figure 2 focuses on the composition of the total implementation costs and time per bed. Table 2 breaks down the costs and time details per bed for each NH.

**Table 2:** Implementation costs, time and resources per NH per bed

Name	Unit per bed	NH1	NH2	NH3	NH4	NH5	NH6	NH7
**PERSONNEL RESOURCES**								
**Organised by research group:**								
Preparatory leadership meetings	Costs (CHF)	56.5	12.55	45.27	39.4	19.96	68.47	14.07
	Time (h)	0.88	0.21	0.91	0.75	0.37	1.31	0.1
Meeting with all NHs	Costs (CHF)	13.24	3.49	23.08	0	0	4.81	12.01
	Time (h)	0.19	0.07	0.38	0	0	0.11	0.12
Leadership and INTERCARE nurses' meetings	Costs (CHF)	59.32	21.36	89.88	45.55	27.02	39.38	31.68
	Time (h)	0.91	0.4	1.8	0.89	0.49	0.72	0.31
INTERCARE nurses' training	Costs (CHF)	340.81	99.24	131.76	158.21	171.05	268.12	0
	Time (h)	7.03	2.48	1.2	3.25	3.42	6.09	0
Phone calls	Costs (CHF)	31.46	10.38	21.62	24.81	13.16	19.25	26.08
	Time (h)	0.65	0.26	0.43	0.53	0.26	0.44	0.36
**Organised by NHs internally:**								
Internal training and information events	Costs (CHF)	89	173.32	48.82	28.79	41.46	271.53	164.95
	Time (h)	1.84	4.23	0.98	0.61	0.75	5.95	0.8
Administration and internal coordination	Costs (CHF)	196.97	217.75	72.41	64.34	266.89	67	339.03
	Time (h)	3.49	3.56	1.45	1.3	4.78	1.31	0.78
**NON-PERSONNEL RESOURCES**								
Travel	Costs (CHF)	19.27	28.66	37.5	49.79	30.54	18.91	18.12
	Time (h)	0.31	0.46	1.07	0.65	1.16	0.34	1.38
Material	Costs (CHF)	0	2.35	0	0	48.97	35.17	27.78
	Time (h)	NA	NA	NA	NA	NA	NA	NA
**Total per NH**	Costs (CHF)	806.57	569.1	470.34	410.89	619.05	792.64	633.72
	Time (h)	15.3	11.67	8.22	7.98	11.23	16.27	3.85

*NA* not applicable

Two NHs did not report any cost, time or resource consumption for "Meetings with all NHs", as they decided not to participate in this strategy. One NH reported no costs for "INTERCARE nurses' training", as this was provided during other formal internal education. Costs, time and resources needed for "phone calls" were influenced both by the number of INTERCARE nurses in the NH and by the length of the NH's intervention period (12–17 months). And even though the "Meeting with all NHs" lasted seven hours for all (Table 1), the NHs' hours/bed ratios differed considerably, as the number of participants per NH varied (average: 4; range: 0–8). The same applies to the "preparatory leadership meeting", which lasted 14 h (Table 1); however, its average number of participants was 6 (range 2–12). Total costs and time per bed were further affected by the number of INTERCARE nurses per bed and their salary.

Regarding material resources, extra materials ranged from none to laptop computers, a printer, voice recording equipment, an additional office, office materials, email accounts, a project information board, literature, pulse oximeters (and/or peak flow meters) and stethoscopes.

### Intervention costs, time and resources

Through the intervention period, the yearly intervention cost—i.e., the INTERCARE nurse salary—averaged 939CHF (range 259–1′513CHF) per bed. The average gross starting annual salary of the INTERCARE nurse was 84′845CHF (range 68′738–97′500CHF). During the INTERCARE study, two NHs made salary adjustments independently of the study, two made no salary adjustment, one paid spontaneous bonuses, and two made adjustments of 100–310CHF increase per year. Depending on the NH, the hourly pay for an INTERCARE nurse was higher than for a regular registered nurse, but less than for a deputy director of nursing. After the study, it was at least as much as for a unit leader.

INTERCARE nurses' age, educational background, advanced training and work experience were considered in their salary classification. They were not funded by the INTERCARE research grant, but via either unit staffing plans or development funds and provisions. Funding through unit staffing plans was drawn from the nursing or physician budget or combined with other general positions’ fee budgets such as site security, management, or supporting staff positions such as quality management.

The average number of INTERCARE nurse per 100 beds was 1.4 (range 0.8–2.0). The average employment percentage per nurse was 76% (range 40–100%). Employment percentages were calculated either as FTEs per number of occupied beds or directly as departmental workload percentages. With one exception, all participating NHs had already hired at least one nursing expert before INTERCARE started; with INTERCARE, that number increased by 0.3–1.0 FTE/100 beds. In our sample, there was no correlation between the number (or FTE) of INTERCARE nurses per bed and the effect of the model.

### NHs' losses and savings due to a hospitalisation

From the NHs’ perspective, the average daily loss of revenue per resident due to a hospitalisation for the years 2017–2020 was 160CHF (range 120–201CHF), while the average daily fixed revenue per resident for the same period was 155CHF (range 130–175CHF). For each hospitalised patient, 100% of NHs' nursing services revenues were lost, alongside an average of 11% (range 0–29%) of hotel services. During each absence, the NH lost an average of 52% of all associated revenues (range 43–61%).

Once a resident was hospitalised, our survey results indicated almost no savings (i.e., reductions of variable costs) for the NHs. Daily savings included several minutes of room cleaning, a lower workload for nursing staff, less laundry, fewer meals and less work for the kitchen. The impact on NHs' costs was minimal. Some savings, e.g., in cleaning materials and food, only become noticeable during several simultaneous hospital stays. However, these remain minimal and not precisely quantifiable. Therefore, for our calculations, we assumed no savings in service costs during a resident's hospitalisation (i.e., that all services costs are fixed).

According to our calculation, between 2017–2020 all NHs showed increases in total daily losses per hospitalised resident. The reasons included the increasing daily revenues from nursing services, which are lost during hospitalisations. For most, daily hotel service losses per resident remained unchanged. The only two exceptions involved changes to the NHs' fee schedules. Fixed revenues differed widely depending on the NHs' different approaches described below.

During the residents' hospitalisation, the reimbursements that the NHs normally receives for nursing services from the health insurance and the Canton are lost. But what happens with the revenue from the resident's share of nursing services and hotel services differed between NHs and years. We observed three different regimes in our sample:

- The resident's share of nursing services and the whole hotel services' revenue was lost, but a reservation fee from the resident was levied.
- Revenue from the resident's share of nursing services was lost, but hotel services' revenue lasted (fixed costs). A resident was receiving "customer credit" or "fee reduction" for not using hotel services.
- The revenue from resident's share of nursing services lasted during the first four days of absence. Hotel services' revenue was ongoing, but in case of hospital stay of more than four days, the resident receives back money for snacks and for the housekeeping.

Because of the NH's confidentiality, no data on the individual NHs' level can be presented in this section.

### Cost-effectiveness analysis

Cost-effectiveness analysis (CEA) showed that, compared to usual/pre-intervention care, the INTERCARE model intervention period was more costly but also more effective: the base-case ICER per avoided hospitalisation was 22′595CHF. The mean additional NH cost during the intervention period was 2′937CHF ± 630CHF per 1′000 nursing days. The average hospitalisation rate fell from 1.27 ± 1.07 per 1′000 nursing days before the intervention period to 1.14 ± 0.93 per 1′000 nursing days during the intervention period.

**Fig. 3:**
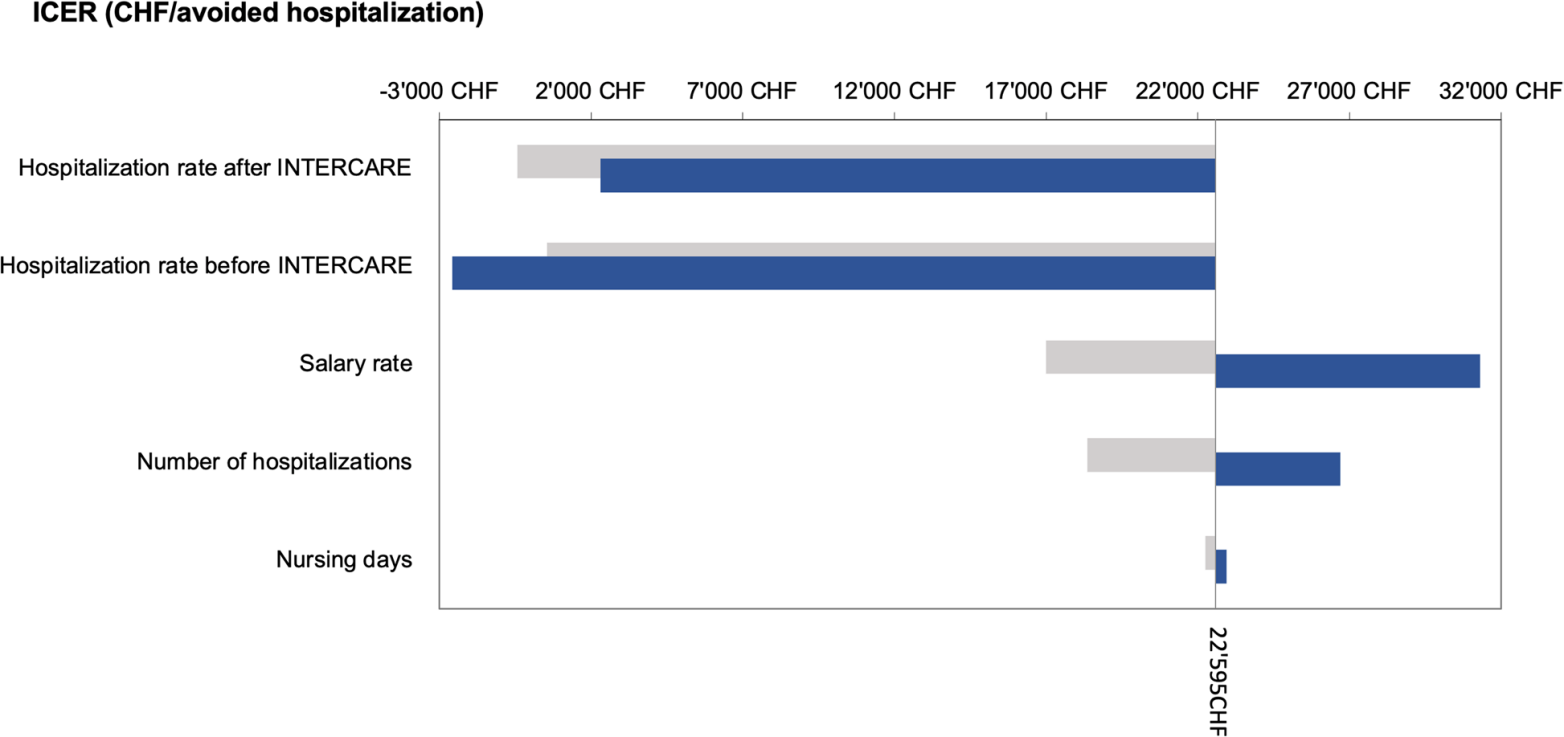
ICER Tornado diagram and detailed results of the one-way sensitivity analysis. The tornado diagram shows results of the one-way sensitivity analysis for the incremental cost-effectiveness ratio (ICER) when the input variable is modified. The vertical line represents the value of the base-case ICER result (22′595CHF/avoided hospitalisation). The grey and blue horizontal bars represent the size of the base-case ICER's change. The grey bars show the change in base-case ICER when there is a 20% increase to the original value or upper limit of the range. The blue bars show the change in the base-case ICER when there is a 20% decrease from the original value or lower limit of the range. E.g., if the salary rate was in its upper limit of the range, the base-case ICER would increase to 31′300CHF/avoided hospitalisation*.* Negative ICER values in our diagram represent the fourth quadrant of cost-effectiveness plane (INTERCARE is dominated) – i.e., incremental costs have positive value and incremental effects negative value

The changes in the hospitalisation rate before and after INTERCARE were the univariate sensitivity analysis variables that most influenced the model. Higher or lower hospitalisation rate after or before intervention respectively led to negative ICER value, where INTERCARE was dominated (i.e., positive incremental cost value and negative incremental effect value). In contrast, changes in nursing days had negligible effects on the model. Figure 3 shows the complete results of our analysis of that model's sensitivity.

## Discussion

The results of our economic analyses showed that, over the entire implementation, the mean INTERCARE implementation action costs and time consumption per bed were 685CHF and 9.35 h respectively, with the possibility to tailor material and personnel resources to each NH’s needs. The average yearly intervention costs (focusing on the INTERCARE nurse) per bed were 939CHF, with the average of 1.4 INTERCARE FTE nurses per 100 beds. The individual employment percentage varied between 40–100%. Residents' hospitalisations were associated with a mean loss of 52% of their NH's normal revenues from them. A CEA showed that the INTERCARE model is more costly but also more effective when compared to usual care, with an ICER of 22′595CHF per avoided hospitalisation.

To date, few studies have economically evaluated the implementation of EBIs in residential long-term care settings [24–26]. Their results highlight how even modest improvements to NHs' clinical and/or nurse practices can lead to significant gains. However, none have provided separate evaluations of the implementation and interventions followed by CEAs. This study is a first attempt to apply a health-economic evaluation in residential long-term care setting fully, with the focus on residents’ hospitalisations.

Our analyses of the INTERCARE implementation strategic actions show high variability regarding costs and hours across NHs (Table 2). Unifying the unit of measurement to per-bed (i.e., instead of per NH) figures allowed clear comparisons across NHs, regardless of size. Local processes, different methods of handling in-house implementation and unpredictable leadership structures likely caused variations in training, meetings, coordination, support and materials. Also, at the start of the implementation, all NHs had different resources available. While all of these elements and differences make it difficult for the managers of a single NH to estimate the economic impacts of introducing the INTERCARE model in their specific organisation, it highlights relevant issues to reflect the complexity of varying real-life practices. In fact, the range estimates and relative values can help NH administrators everywhere, by illustrating what percentages of costs apply to the various implementation actions and how these can vary. (For Swiss settings, we also presented absolute values.) Based on our variation findings, managers should plan sufficient time for internal training sessions and informational events in order to avoid the potential delays.

In practice, inadequate project controls and resource management often result in cost, time and resource overruns [27, 28]. From the studied NHs' perspective, flexibility regarding resources and implementation strategy actions allowed NHs to minimise implementation time and costs. The largest potential implementation cost reductions were in “Administration and internal coordination”, “Internal training and information events”, and “INTERCARE nurse training”. Together, they accounted for 78% of all implementation costs and 73% of all implementation time. The first two represent a need among NHs for support streamlining their internal approaches to innovation—not only regarding implementation strategies per se, but also in finding more efficient processes, e.g., of training staff and coordinating work internally.

The intervention costs, time and resources needed also varied broadly across NHs, as well as how the INTERCARE nurse was included in the organization. We had a minimum requirement of 0.6 FTE/80 beds, but beyond that, it is difficult to conclude that there is no correlation between the number (or FTE) of INTECARE nurses per bed and impact. All INTERCARE nurses focused on coaching and supporting care teams with noted differences in additional tasks they performed (e.g., quality improvement, conceptual work). Moreover, based on Guerbaai et al. ([29] in preparation), the impact of the INTERCARE model is mainly the result of using the two core components, evidence-based instruments and advance care planning, where the INTERCARE nurse was key in introducing it and supporting the implementation.

In cases where NHs evolved existing positions (i.e., nursing experts hired before INTERCARE) to intervention roles, additional implementation costs were considerably lower. Thus, we strongly recommend that every NH builds on internal resources, selecting experienced registered nurses who can be trained as INTERCARE nurses. Within NHs, the implementation of INTERCARE nurse positions represents a sustainable decision—one grounded in evidence-based medicine and implementation science. INTERCARE nurses will both strengthen NHs’ long-term care effectiveness and reduce hospitalisation-related losses. While supplying clinical nursing expertise fundamental to high-quality care [30], they improve interprofessional communication, which is a global healthcare priority [31]. Moreover, working with other core elements of the INTERCARE model, INTERCARE nurses ensure the timely provision of expertise and continuity of medical care and improve the transparency of related care processes and structures. In addition to improving patient care, this increases NHs' attractiveness for potential employees.

Hospital care normally costs considerably more than NH care [32] and it exposes residents to adverse events and complications that often accompany hospital stays [33–35]. Even temporarily transferring residents from their familiar surroundings creates an additional burden for them [36]. Thus, most studies of health care utilisation, costs and savings are from the healthcare payer perspective [36–38]. However, to facilitate efficient care provision for NH's residents, it is also essential to understand the NHs’ perspective. For the NH, a resident's hospitalisation implies two financial outcomes: i) the loss of variable revenues for nursing and hotel services; and ii) the saving of variable costs for hotel and nursing services. Our cost analysis showed almost no savings for NHs during residents' hospitalisations, but confirmed that for NHs, hospitalisation costs are also much lower than for healthcare payers [36–38].

Even the uses of revenues from nursing and hotel services varied widely between studied NHs, all NHs lowered their fees for residents during hospitalisation. For that period, they also lost all the revenues arising from those residents' health insurance, their cantons and municipalities. In our analyses these losses of revenues varied in total from 120–201CHF per resident and day, which represented 43%–61% loss of the revenues associated with those patients. Thus, reducing hospitalisations would greatly benefit not only the healthcare payers (by removing hospitalisation costs), but also NHs (by avoiding revenue loss).

Our CEA findings further support the evidence that decreasing NH residents' hospitalisations decreases healthcare expenditures [39–41]. In Switzerland, the estimated mean cost of usual care at a university hospital is 5′530CHF/day, with a mean total cost of 41′158CHF/hospitalisation [42]. Narrowing the scope, the mean cost of university hospital stays due to ambulatory care-sensitive conditions is 13′267CHF [37]. Our CEA shows that, compared to standard care, the INTERCARE intervention in NHs costs 22′595CHF per avoided hospitalisation. This represents net financial costs to the NH management for an extra effect. As no official WTP (willingness to pay) in NHs exist, we cannot state, whether our result is cost-effective. However, our analysis suggests a possible future development: Budget impact analysis (BIA). For BIA, additional detailed data needs to be collected.

In a CEA, the ICER is calculated by dividing the difference in total costs (incremental cost) by the difference in the chosen measure of effect (incremental effect) [43]. Thus, higher incremental effects lead to lower ICER results. For our calculation, we choose the hospitalisation rate as an effect. While short ambulatory/outpatient hospital visits represent a significant part of all NH residents' hospital stays [44], data limitations allowed us to include only hospitalisations with at least one overnight stay. As these short ambulatory/outpatient visits are often preventable [36, 45, 46], including them in the calculation would lead to lower ICER. Therefore, one recommendation from this CEA would be for NH leaders to start properly monitoring residents' short hospital visits. Only by doing so can they accurately monitor the effects of hospitalisation-reducing activities.

It is also important to mention that each CEA can consider only a single effect [47]. However, the INTERCARE model's effects extend well beyond lowering NH residents' hospitalisation rates: as an intervention, it supports care workers in daily practice, bolsters their confidence, and it prevents residents from experiencing health crises. As Basinska et al. [8] showed, such effects are urgently needed in NHs.

By applying INTERCARE's core intervention elements, the new care model is increasing NHs' capacity for early recognition and treatment of residents’ health changes. This provides smoother workflow and reduces additional NH resource needs regarding new admissions and readmissions, neither of which we included in our calculation. Moreover, NHs who implemented the INTERCARE model reported intangible benefits, e.g., increased attractiveness for potential employees, more effective communication and collaboration between teams and inter-professional groups, reductions in numbers of physician visits, decreased use of mobile physician teams during nights and weekends and increased satisfaction among residents and their relatives. Considering these subjective values of residents, their families and NH staff, together with our health-economic results, we strongly recommend that NHs implement the INTERCARE model.

Several limitations must be considered in generalising our results. This study included a relatively small number of NHs. Another frequently reported hospitalisation avoidance programme, INTERACT II, included 25 NHs and has been operationalised in Canada, the United Kingdom and Singapore [44, 48, 49]. As the INTERCARE programme included only 11 NHs from the German part of Switzerland, its results are less robust. However, preparation for the INTERCARE follow-up study, which will include a higher number of NHs, is already underway. This will allow us to observe in more detail the correlation between the different policies of the NHs (billing during the resident's hospitalisation, way of INTERCARE implementation) and the effect of the intervention and thus draw more targeted recommendations. Moreover, it will help to overcome other limitations of the ICER: its high sensitivity regarding the hospitalisation rate variable, its inability to consider other effects and the lack of probabilistic sensitivity analysis. Further, the follow-up study team will have more time to deal with the above-noted lack of resident outpatient visit data (less than one overnight stay), which distorted the current study's results by underestimating the effectiveness of INTERCARE model. I.e., this health economic analysis' ICER skews high. Additionally, the shortage of detailed information in our TDABC report prevented cost composition analysis by study period, as several NHs did not provide specific dates for all implementation strategy actions. It is also important to mention that the current lack of standardised measures and guidance is identified in implementation science as a critical impediment to high-quality, implementable findings [50].

## Conclusion

While many factors require consideration regarding implementation of the new care model, this health-economic evaluation will help to determine its relative efficiency [51] regarding a wide range of contexts. The results showed that the INTERCARE model is more costly but also more effective when compared to usual care. The introduction of nurses in expanded roles—particularly INTERCARE nurses—and the implementation of the other core INTERCARE elements are EBIs that will benefit not only residents, but also their families, NH staff and NHs themselves. Our findings indicate that the investments necessary to establish the INTERCARE nurse position and implement the associated care model yield sustainable and potentially cost-effective improvements of care with respect to individual NH’s WTP. Further implementation and evaluation of the INTERCARE model via randomised controlled studies are planned to build a stronger evidence base regarding its clinical and economic effectiveness.

In addition to the findings this paper reports, it fills a hitherto unmet need to evaluate implementation strategies' effectiveness [4, 52]. Such evaluations are critical steps toward increasing healthcare efficiency by promoting successful uptake of EBIs. Thus, this study not only informs NHs administrators, potential funding sources and policymakers about INTERCARE's costs and benefits; it also offers clear insights into how to conduct economic analyses of implementation strategies and interventions in real-world settings. By doing so, it strengthens the basis for cost-based comparisons between implementation strategies and intervention elements designed to reduce NH resident hospitalisations.

## Data Availability

While the data upon which this study's findings are based are available from participating NHs, restrictions apply to their availability. I.e., they were used under license for the current study, and are not publicly available. However, data directly relevant to our findings are available from the authors upon reasonable request and with permission from the participating NHs.

## Abbreviations

BIA: Budget impact analysis
CEA: Cost-effectiveness analysis
CHF: Swiss Franc
EBI: Evidence-based intervention
ERIC: The Expert Recommendations for Implementing Change
FTE: Full-time equivalent
ICER: Incremental cost-effectiveness ratio
ISPOR: The Professional Society for Health Economics and Outcomes Research
NH: Nursing home
TDABC: Time-driven activity-based costing
WTP: Willingness to pay
